# Cytokine Gene Polymorphisms Modulate Isohemagglutinin Titers and Classes: Another Aspect Towards the Link Between ABO Groups and Human Pathologies?

**DOI:** 10.3390/ijms27083629

**Published:** 2026-04-18

**Authors:** Letizia Scola, Daniele Magro, Chiara Motisi, Alessia Di Salvo, Matteo Bulati, Chiara Bellia, Carmela Rita Balistreri

**Affiliations:** 1 Cellular and Molecular and Clinical Pathological Laboratory, Department of Biomedicine, Neuroscience and Advanced Diagnostics (Bi.N.D.), University of Palermo, 90134 Palermo, Italy; letizia.scola@unipa.it (L.S.); daniele.magro@unipa.it (D.M.); chiara.motisi1@gmail.com (C.M.); alessiads9834@gmail.com (A.D.S.);; 2 Transfusion Medicine Unit, University Hospital “Paolo Giaccone”, 90127 Palermo, Italy; 3 Istituto Mediterraneo per i Trapianti e Terapie ad Alta Specializzazione and Istituto di Ricovero e Cura a Carattere Scientifico, 90127 Palermo, Italy

**Keywords:** ABO antigens, anti-A and anti-B isohemagglutinins, titers and classes, intra/interindividual variability, functional polymorphisms, cytokine genes

## Abstract

Anti-A and anti-B antibodies are essential for monitoring adverse reactions in organ transplants and transfusions. However, their importance is also growing due to their involvement in the pathophysiology of various human diseases, such as infections, although this is currently the subject of heated debate. A characteristic heterogeneity in the titers and classes of anti-A and anti-B antibodies is observed among individuals. Several factors appear to be responsible, such as everyone’s specific immune profile, age, sex, microbiota composition, lifestyle, and health status. The immune profile, the result of a specific genetic predisposition and mediated and controlled by cytokines, shows a bidirectional relationship with ABO antigen expression, the gut microbiota, and the levels and class switching of anti-ABO antibodies. Associations between ABO groups and circulating levels of cytokines and chemokines further highlight this complex interaction. To better understand the role of the immune profile in this context, we evaluated, for the first time, the possible association between polymorphic variants in the regulatory regions of the genes encoding the cytokines IL-18, IL-1, IL-4, IL-6, IFN-γ, and IL-10 and anti-A and anti-B antibody titers and classes by group and in total. We also assessed the levels of these cytokines in each group and their correlations with anti-A and anti-B antibodies, as well as with age and associations with gender. Significant data were obtained that may contribute to a better understanding of the other roles of ABO antibody titers.

## 1. Introduction

Accumulating evidence in the literature suggests that ABO isohemagglutinins production in everyone is the result of a complex bidirectional interaction between the microbiome/microbiota, T- and B-cell immune subsets, and genetic factors [1,2,3,4,5,6,7,8]. Accordingly, some researchers have tested 2801 microbial taxa against 7,967,866 human genetic variants and identified genome-wide associations for 471 taxa, including the *ABO* locus, which has been shown to drive diverse microbes within the microbiome [1,2,9,10,11,12]. In addition, everyone’s immune system interacts in a complex manner with blood group antigens and the gut microbiota, influencing isohemagglutinin titers, classes, and switching [13,14,15,16,17,18,19,20]. However, everyone’s immune profile is the result of a specific genetic background [21,22,23,24,25], which can likely influence isotype switching and antibody titers, i.e., the quantitative and qualitative expression of natural and immune antibodies [26,27].

In line with this, it is known that IL-18, IL-1, IL-4, IL-6, IL-13, IFN-γ, and IL-10 are involved in the production and release of natural and immune-mediated anti-A and anti-B antibodies, and single nucleotide polymorphisms (SNPs) in their gene’s sequences have been identified to modulate their circulating levels, and consequently the humoral immune responses [28,29]. IL-18 belongs to the IL-1 family and, in addition to inducing IFN-γ production and a TH1 lymphocyte response, promotes the secretion of natural antibodies in peritoneal B1 lymphocytes and splenic marginal zone lymphocytes [30]. Two SNPs have been detected in the promoter region of the IL-18 gene, at −607(C/A) *rs1946518* and −137(G/C) *rs187238*. Considering the functional significance of these two SNPs, it has been shown that the presence of cytosine at −607 from the transcription start site and the presence of guanine at −137 from the transcription start site have been associated with increased mRNA synthesis and increased IL-18 production [31]. IL-1 plays a role in promoting inflammatory and adaptive responses [32], and SNPs in its receptor, as well as the IL-1R *rs2234650*, have been shown to modulate antibody titers. Regarding IFN-γ, which can induce a TH1 lymphocyte response, the polymorphic variant in the IFN-γ gene, +874A/T, has been observed to modulate antibody titers, as well as the polymorphic variant *rs2834213* in the *IFNγR2* gene encoding the IFN-γ receptor. IL-6 is a proinflammatory cytokine that plays a significant role in promoting humoral immunity. The *rs1800795* SNP is known to influence IL-6 levels; in particular, the main −174G allele is associated with an increase in mRNA expression, up to 2.4-fold following IL-1 stimulation, while the C allele is associated, in the homozygous or heterozygous state, with low IL-6 production. Like IFN-γ, which is the key cytokine for the isotype switch from the μ chain to the γ chain, i.e., from IgM to IgG, IL-4 stimulates the proliferation, maturation, and differentiation of B lymphocytes into plasma cells that actively secrete IgE and IgG4 [33,34]. Furthermore, IL-4 is essential for the maintenance of naive B cells and the production of memory B cells after exposure to a natural or vaccine antigen [35]. Regarding IL-10, it has been shown that the *rs800871C* allele is associated with increased cytokine production, and the homozygous genotype is associated with a greater reduction in the inflammatory response with risk of HBV infection [36] and risk of SLE [37]. Concerning these cytokines, IL-13 is a critical immunoregulatory cytokine produced by activated Th2 cells, and diverse SNPs modulate its expression. The *IL-13* SNP rs1800925 is a common SNP in the promoter region, which caused C to T transition at position − 1112/1024/1055, and determines an increased expression of such cytokine [38,39,40,41,42,43,44,45,46]. The TNF-α cytokine is also involved in the production of antibodies, and the rs1800629 (−308G > A) SNP in the promoter region enhances the expression and levels of TNF-α.

These observations sparked our interest in investigating, in this study, whether SNPs in the genes encoding the cytokines described above (see Table 1) could contribute to influencing isohemagglutinin titers and classes. To this aim, 108 donors were recruited from the Immunohematology and Transfusion Medicine Unit of the Paolo Giaccone University Hospital and from municipal and provincial Avis centers in the province of Palermo. The average age of the donors was between 30 and 50 years, and there were 64 men and 44 women with the following ABO blood groups: 34 with group 0 (19 men and 15 women); 41 with group A (28 men and 13 women); and 33 with group B (20 men and 13 women). All selected SNPs were genotyped (see Table 1), and the titers and classes of anti-A and anti-B antibodies were assessed, as well as their associations. We also assessed the levels of these cytokines in each group and their correlations with anti-A and anti-B antibodies, as well as with age.

## 2. Results

### 2.1. Titration of Total Isohemagglutinins of the IgM Anti-A and Anti-B and IgG Anti-A and Anti-B Classes

Titration of total isohemagglutinins of the anti-A and anti-B IgM and anti-A and anti-B IgG classes was performed on all the samples examined. The median of the titers was calculated by using GraphPad Instat 3.1 software (see Tables S1–S10 in Supplementary Materials).

### 2.2. Associations Between Titers Above or Equal to the Median and Cytokine Polymorphisms in Each Blood Group and in Total

Associations between titers above or equal to the median and below the median with the cytokine gene SNPS were assessed. First, the analysis was executed by stratifying for each group (see Table 2, Table 3 and Table 4) and considering only those cytokine polymorphisms for which a significant association was obtained in the overall assessment. However, the specific group analysis was based on small numbers and, therefore, in some cases did not provide significant data. This led us to evaluate the eventual association of global anti-A and anti-B IgM and IG titers with the cytokine SNPs (Table 5 and Table 6). Some significant associations were observed.

### 2.3. Levels of Cytokines in Three ABO Groups and Their Correlations with Anti-A and Anti-B IgG Antibodies Titers

**Figure 1:**
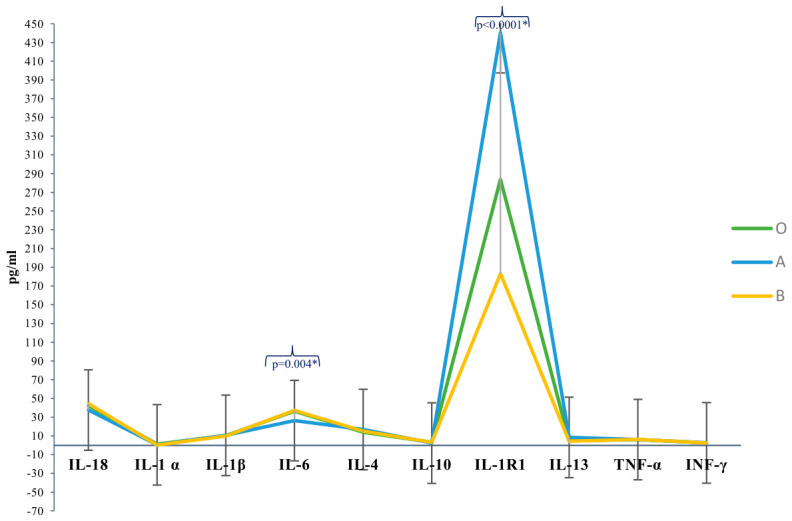
Levels of circulating cytokines in pg/mL in plasma samples from ABO donors * *p* values were detected by using ANOVA test corrected by Bonferroni.

Figure 1 reports the circulating levels of the cytokines selected in the study from the three ABO groups. We detected significant differences between the three groups in the levels of IL-6 and IL-1R1. This led us to assess eventual correlations with the titers of all anti-A and anti-B IgG antibodies and cytokines levels and demographic factors, i.e., age and gender. As shown in Table 7, we assessed a significant negative correlation with age and the circulating levels of IL-10 by evidencing a significant biological effect of such factors on both anti-A and anti-B IgG antibodies. In contrast, a positive correlation was determined between anti-A and anti-B IgG antibodies and IL-6, IL-1R1, INF-γ, and IL-4 levels.

### 2.4. Binary Regression Analysis of Potential Factors Associated with Titers of Anti-A and Anti-B IgG Antibodies

By using binary logistic regression analysis, we evaluated the independent factors that were significantly associated with the titers of all IgG antibodies. We interestingly observed that increased age, smoking, and levels of IL-6, IL-1R1, INF-γ, and IL-10 were significantly associated with the titer of such antibodies (see Table 8). Surprisingly, in the binary logistic regression analysis adjusted for age and levels of IL-6, IL-1R1, INF-γ, and IL-10, we detected that age and IL-6, IL-4, IL-10, and IL-6rs1800795C/IL-4rs2243250T/IL-10rs3021097T/IFN-γrs2430561T combined genotype profile were the unique independent risk biomarkers associated with the titers of such antibodies (see Table 8). Accordingly, the Hosmer–Lemeshow test statistic was 7.12 (df = 7.9, *p* = 0.52), which revealed good model fit.

## 3. Discussion

IL-18, IL-1, IL-4, IL-6, IFN-γ, and IL-10 are involved in the production and release of natural and immune-mediated anti-A and anti-B antibodies, and SNPs have been identified in their gene sequences, modulating their circulating levels and, consequently, humoral immune responses. Consistent with this, we assessed whether SNPs in the genes encoding such cytokines contribute to influencing isohemagglutinins titers and classes. The analysis of the results obtained demonstrated that the *−607 GG* (minor producer) genotype of the *IL-18 rs187238* SNP was less frequent in our population of healthy donors with high anti-A IgM titers, and this trend was maintained even when performing a group-specific analysis. Consistent with these data, we found a higher frequency of the *−137 GG* genotype of the *IL-18 rs1946518* SNP in subjects with anti-B IgG titers, higher than that found in subjects with non-elevated titers, who instead had a higher frequency of heterozygosity. This result was also confirmed by group-specific analysis in group O, both for the GG genotype (*p*: 0.0155, OR: 3.083, CI: 1.255–7.577) and for the heterozygous GC genotype (*p*: 0.0054, OR: 0.2487, CI: 0.09588–0.6450).

Regarding the *IL-1R1 rs2234650 SNP*, a significant contribution was made by anti-B IgM. Specifically, the *CC* genotype of *rs2234650* is associated with lower antibody titers, as also observed in a previous study [47]. Since the presence of “Thymine” in *rs2234650* has been shown to correspond to a reduced expression of the IL-1 receptor R1 [47], we can assume that a moderate inflammatory response is sufficient to influence humoral immunity. Furthermore, we observed a significantly elevated association between anti-A IgG titers and the polymorphic variant in the *rs2834213* SNP of the *INF-γR2* gene (INF-γ receptor), whose minor allele (G) is associated with a higher expression of the receptor [48]. However, no significant data emerged regarding the association with the polymorphic variant of the *INF-γ* gene (*+874A/T*). Since higher anti-A IgG titers correspond to the low-production “*INF-γRB2 AA*” genotype, our results suggest that increased receptor expression is not necessarily correlated with higher IgG titers [49]. Indeed, lower antibody titers are associated with the wild-type genotype, but especially with the heterozygous genotype, which is typical of limited receptor production. The association persists, although borderline statistically significant (given the insufficient number), also in group “B” but not in group “O.” The significance of this association is unclear but, on the other hand, the virtual influence of this ‘intronic SNP’ (intron 2) is also complex, since it is not located in proximity to a splice site (in fact, it is found 5582 nucleotides downstream of a splice donor site and 877 nucleotides upstream of a splice acceptor site) but, on the other hand, it is in linkage disequilibrium with three SNPs contained in a 300 bp promoter segment, forming a haplotype associated with high transcriptional activity in vitro [50]. We also observed that the wild-type allele *rs1800795G IL-6* SNP in homozygous or even more so in heterozygous state is linked to a sustained production of isohemagglutinins, also observing group-specific production, which confirms the role of IL-6 in the maintenance and qualitative remodeling of natural antibody production [51].

In subjects with higher total anti-B IgG titers, a higher frequency of heterozygosity for the IL-4 polymorphism at *−590C/T* was found [52]. Therefore, a genotype associated with limited IL-4 production [53] could contribute to the maintenance of optimal IgG production. Regarding IL-10, we detected in donors with low anti-B IgM and anti-A IgG antibodies titers a higher frequency of the heterozygous *rs800871* genotype. This result was not confirmed by group-specific analysis for anti-A IgG, but the significance remained for anti-B IgM in group “A”.

## 4. Materials and Methods

### 4.1. Population Selected in the Study

For this study, blood samples were collected from 108 healthy donors attending the Immunohematology and Transfusion Medicine Service at the Paolo Giaccone University Hospital and from Avis centers. The average age was between 30 and 50 years, 64 males and 44 females: 34 were blood group O (19 males and 15 females); 41 were blood group A (28 males and 13 females); and 33 were blood group B (20 males and 13 females). This study was approved by the Ethics Committee at the Affiliated Hospital of the University of Palermo (2024-UP-080424).

The blood samples were collected at the time of donation, made incoagulable with EDTA, and stored at −20 degrees Celsius. To evaluate isohemagglutinins, the Neo Iris analyzer from Immucor/Werfen (Norcross, GA, USA) was used, while to evaluate polymorphisms, an application of the Polymerase Chain Reaction (PCR), namely Kaspar PCR, was used, as reported in the subsequent paragraphs.

### 4.2. Evaluation of the Titers of Isohemagglutinins with Neo Iris Instrument

This assessment was performed by using the Immucor Neo Iris instrument. Blood samples were inserted into the instrument and brought to room temperature. The instrument performs a fully automated titration, that includes several steps: (a) it performs serial dilutions of the entire samples; (b) it tests Coombs dilutions for IgG and direct agglutination for IgM; (c) the samples are then tested against known cells, and the final titer is reported as the reciprocal of the last dilution that produced a positive result; (d) finally, it measures the antibody’s strength, which is the result of its avidity and concentration. For this study, low-titer IgG and IgM were assessed as follows:

- IgM anti-B and IgG anti-B for blood group A
- IgM anti-A and IgG anti-A for blood group B
- IgM anti-A and anti-B and IgG anti-A and anti-B for blood group O

Typically, a low-titer test is performed first, and if this is positive, a high-titer assessment is performed.

### 4.3. DNA Sample Extraction

The QIAGEN kit was used to extract DNA from peripheral whole blood. Before starting the protocol, the peripheral whole blood samples, previously frozen at −20 °C, were brought to room temperature. The two wash buffers included in the kit were then prepared by adding 100% ethanol in the following quantities: −25 mL to the bottle containing Wash Buffer 1 (AW1); −30 mL to the bottle containing Wash Buffer 2 (AW2). The wash buffers are used to remove any contaminants (proteins and other cellular debris) from the sample. The lysis solution was prepared by adding 20 µL of PK, 200 µL of blood sample, and 200 µL of lysis buffer (AL) to the lysis tube. The lysis solution, which disrupts the plasma and nuclear membranes, was then mixed by vertexing for 15 s, incubated at 56 °C for 10 min, and then centrifuged for 5 s at maximum speed. A total of 200 µL of 100% ethanol was added to the lysis tube and mixed by vertexing for 15 s. Ethanol allows the genetic material to precipitate. The contents were then transferred to a centrifuge column and centrifuged at 8000 rpm for 1 min then transferred to a clean wash tube. A total of 500 µL of AW1 was added to the wash tube. The mixture was centrifuged at 8000 rpm for 1 min then transferred to a second wash tube. A total of 500 µL of AW2 was added to the wash tube and centrifuged at 14,000 rpm for 1 min. The entire contents were then transferred to another wash tube, and the centrifugation was repeated at maximum speed for 3 min to completely dry the membrane. The entire contents were transferred to a 1.5 mL elution tube, and 200 µL of elution buffer was added to the center of the membrane. The membrane was incubated at room temperature for one minute. The eluate was centrifuged at 8000 rpm for one minute to obtain the eluate in the 1.5 mL tube. The DNA was then frozen at −20 °C.

### 4.4. Genotyping by the KASPar (Competitive Allele Specific PCR) System

The KASPar (Competitive Allele Specific PCR) system was used to study polymorphisms in extracted DNA. This method uses specific primers and fluorescent signals to identify SNPs. Therefore, for each polymorphism to be studied, the samples and primers were brought to room temperature before starting the method. The amplification mixture was then prepared in a 1.5 mL tube by adding:

- H_2_O to dilute the components, ensure uniform distribution, and achieve the desired final reaction volume (to maintain reagent concentrations)
- primers, which are short nucleotide sequences that, by binding to specific DNA sequences, provide a starting point for DNA polymerase
- a mixture containing the thermostable DNA polymerase TAQ polymerase (an important enzyme for the synthesis of new DNA strands), dNTPs (the building blocks for strand synthesis), magnesium chloride (required for DNA polymerase activity), the fluorochromes FAM and VIC, a passive reference fluorochrome (ROX), and a buffer to maintain reagent stability. The ROX serves to normalize the fluorescent signals emitted by the fluorochromes FAM and VIC.

To each well of the plate, 1 µL of each previously extracted DNA sample was added, followed by 7 µL of the mixture. The plate was placed in the 7300 Real-Time PCR System to read the initial fluorescence. Subsequently, for amplification, it was inserted into a standard 2720 thermal cycler, which uses repeated heating and cooling cycles to promote amplification. The three steps that make up a cycle are denaturation, annealing, and elongation. The cycles are repeated 30 times. The KASPar method uses the following program:

Step 1—94 °C for 15 min, the temperature required for DNA strand denaturation and enzymatic activation of TAQ polymerase

Step 2—94 °C for 20 s

Step 3—65–57 °C for 60 s

Steps 2 and 3 are repeated for 10 cycles and correspond to the annealing phase in which the primers bind to their complementary region (which corresponds to the region to be amplified), serving as a reference point for DNA polymerase to form new molecules.

Step 4—94 °C for 20 s

Step 5—57 °C for 60 s

Steps 4 and 5 were repeated for 26 cycles and consisted of denaturation and annealing. Finally, the plate was returned to the 7300 Real-Time PCR System for final fluorescence detection at 4 °C. SDS vs 1.3 software was used to analyze the results, which plots the fluorescence intensity of FAM and VIC fluorochromes on the X and Y axes, respectively.

### 4.5. Cytokines

By using serum blood samples from 56 donors (24 of 0 group, 19 of A group, and 13 of B group), levels of IL-18, IL-1α, IL-1β, IL-6, IL-4, IL-1R1, IL-13, TNFα, IFN-γ, and IL-10 were measured by a single laboratory on Luminex fluorescent bead-based assay panels (n. EPXR450-12171-901, ThermoFisher, Scientific (Waltham, MA, USA); Bender MedSystems GmbH, Campus Vienna Biocenter 2, Vienna, Austria). Assays were run at a centralized laboratory according to manufacturer protocol, concentrations were determined with four- or five-parameter standard curves, and samples were run in duplicate to calculate average measurements.

### 4.6. Statistical Analysis

Total isohemagglutinin titers for IgM anti-A, anti-B, and IgG anti-A and anti-B were measured on all samples. The median titer was calculated by using the Instat GraphPad version 3.06 software (GraphPad, San Diego, CA, USA). The association between titers above and equal to the median and those below the median with cytokine polymorphisms was assessed by using Fisher’s exact test. Subsequently, the analysis was repeated, stratifying by individual group, by considering only the cytokine polymorphisms for which, in the overall assessment, a significant association was obtained. The analysis was then repeated, stratifying by male and female gender. These data were reported in Supplementary Materials.

Allele frequencies and genotype distributions were assessed by gene counting using an online statistical analysis tool for SNP evaluation (https://www.snpstats.net/start.htm, accessed on 18 December 2025). Data were tested for goodness of fit between observed and expected genotype frequencies, according to Hardy–Weinberg equilibrium, using the Pearson distribution and χ2 tests. Significant differences in allele, homozygous, and heterozygous genotype distributions between groups were calculated using Fisher’s exact test (adjusted for age and sex). Odds ratios (ORs), 95% confidence intervals (95% CIs), and *p*-values (*p*-value cutoff < 0.05) were determined using GraphPad InStat version 3.06 software (GraphPad, San Diego, CA, USA). The correlations between two continuous variables were assessed with Pearson’s test or non-parametrical Spearman correlation test. Differences in the cytokines levels between two groups were evaluated using the Kolmogorov–Smirnov test or the Student *t*-test when appropriate, while one-way ANOVA or Kruskal–Wallis tests followed by Bonferroni correction were applied to compare more than two groups. Binary logistic regression analysis was used to find the possible independent associations between the age, cytokines levels, and the IL-6rs1800795C/IL-4rs2243250T/IL-10rs3021097T/IFN-γrs2430561T combined genotype profile. The Hosmer–Lemeshow test was used to check goodness-of-fit of the logistic regression. *p*-values are two-sided, and values <0.05 were considered statistically significant.

## 5. Conclusions and Limitations

The limited number of donors in the cohort-specific analysis makes it difficult to accurately interpret the data obtained. However, they suggest that the immunological microenvironment, and in particular cytokine levels, significantly influence isohemagglutinins secretion. Specifically, our data indicate that moderate inflammatory conditions can influence the production of natural antibodies, which in turn are influenced by other factors, such as age. The assessed correlations showed that age negatively modulates isohemagglutinins titers and represents one of the independent factors significantly influencing these titers. On the contrary, the levels of IL-6, IL-4, IL-10, and IFN-γ correlate positively with the titers of IgG isohemagglutinins and represent, together with the *IL-6rs1800795C/IL-4rs2243250T/IL-10rs3021097T/IFN-γrs2430561T* combined genotype profile, the independent factors that significantly affect the titers of these antibodies and especially of all IgG. Concerning the gender, no significant data were obtained (see Supplementary Materials). In the population enrolled in our study, the woman number was much smaller and unbalanced with respect to the number of men, and consequently the comparison was not significant. However, the literature evidence underlines the impact of gender, as well as of environmental factors, such as diet, infection and related vaccinations, on ABO antibody titers [54,55]. A vegetarian diet has been reported to show a significant association with elevated titers [55]. Women have been reported to have higher antibody titers than men due to pregnancy [56], but in our study, the female population is insignificant in its number.

This study also demonstrates that our donor population presents a predominance of low ABO isohemagglutinin titers during the study period (80% or higher) for both IgM and IgG antibody titers compared to previous studies [55,56,57]. Furthermore, IgG titers are higher than IgM titers for both anti-A and anti-B isohemagglutinins, as previously reported in the literature [58]. In a similar study, the critical cutoff value for isohemagglutinin titers was estimated to be 128 for IgG and ≥64 for IgM [59]. The authors suggested that IgM titers 64 (assessed by the direct hemagglutination method) and IgG titers ≥128 (tested in solid phase on a NEO IRIS automated immunohematology analyzer) should be considered high titers. We also obtained higher IgG titers in group O. Certainly, defining critical titers could be useful in formulating institutional guidelines on ABO-incompatible platelet transfusions and in the management of ABO-incompatible solid organ transplants.

Our results highlight the imperative need for further studies on a much larger number of donors, which could eliminate the issue of non-significant associations of antibody titers with selected SNPs, as well as the question on low titers, as above discussed. Furthermore, the study should not only take gender differences into account but also other factors, such as ABO type and genotypes, which, together with sex, age, and ethnicity, contribute to modulating natural antibody titers, as recently highlighted by Jacob et al. [60].

However, the significant associations observed lead us to suggest that in blood centers that do not have automated and routine titer screening procedures, these results could be used as a method to efficiently identify donors with low titers a priori. Therefore, genotyping for cytokine SNPs should also be included in donor screening. However, this highlights the need to further clarify the role of this association demonstrated in this study, by using a multi-centers donor population, thanks to the collaboration with all transfusion centers, at least in Sicily. Furthermore, standardization of methodologies would be necessary, since it influences the titer of isohemagglutinins, as well as verifying whether the association is stronger with anti-A and anti-B immune antibodies or with natural ones, given the large number of samples. Here, the association was more significant by evaluating the titers of all the anti-A and anti-B IgG antibodies, natural and immune.

Much remains to be demonstrated, but the data are encouraging. Thus, this study should be considered preliminary. Increasing the donor samples for re-evaluation will be useful, as will confirming the group-specific/gender and age analyses. Therefore, it appears possible to delineate gene profiles associated with the ABO system and able to influence high or low isohemagglutinin titers, which are important for transfusion reactions and ABO-incompatible transplants and predict risk for diseases.

## Figures and Tables

**Table 1:** SNPs analyzed and their characteristics.

Gene	SNP	Position	Minor Allele	Biological Effect	References
IL-1α	rs1800587	2:112785383	T	The minor allele is associated with a greater production of the cytokine	[21]
IL-1β	rs1143634	2:112832813	T	The minor allele is associated with greater production of the cytokine	[22]
	rs16944	2:112837290	A	The minor allele is associated with a reduced production of the cytokine	[23]
IL-1RN	rs315952	2:113132727	C	The minor allele is associated with an increased efficiency in inflammation control	[24]
IL1-R1	rs2234650	2:102141867	T	Alleles create two alternative putative binding sites for two different transcription factors	[25]
IL-18	rs187238	11:112164265	G	The minor allele is associated with greater production of the cytokine	[25]
	rs1946518	11:112164735	T	The minor allele is associated with a reduced production of the cytokine	[26,27]
IL-6	rs1800795	7: 22727026	C	The minor allele is associated with a reduced production of the cytokine	[28]
TNF-α	rs1800629	6:31575254	A	The minor allele is associated with a greater production of the cytokine	[29]
IL-10	rs1800896	1: 06773552	G	The minor allele is associated with greater production of the cytokine	[30]
	rs1800872	1:206773062	A	The minor allele is associated with a reduced production of the cytokine	
	rs3021097	1:206773289	T	The minor allele is associated with a reduced production of the cytokine	
IL-4	rs2243250	5:132673462	T	The minor allele is associated with a greater production of the cytokine	[31]
IL-13	rs1800925	5:132657117	T	The minor allele is associated with a greater production of the cytokine	[33]
IFN-γ	rs2430561	12:68158742	T	The minor allele is associated with a greater production of the cytokine	[33,34]
IFNγR2	rs2834213	21: 3420603	G	The minor allele is associated with a greater production of the cytokine	[34]

**Table 2:** Association between titers of IgM and IgG anti-B of group O, above or equal to the median and below the median, and cytokine polymorphisms.

Blood Group O							
**IgM anti A**							
*IL-18-607GT rs1946518*	Ab titer ≥ 8%	Ab titer ≥ 8%	Ab titer < 8%	Ab titer < 8%	OR	CI	*p* value *
GG	14	0.625	7	1	0.08406	0.004329–1.632	0.0664
GT	10	0.312	0	0	10.161	0.5220–197.81	0.0694
TT	1	0.063	0	0	0.9184	0.03373–25.006	NS
**IgG anti-A**							
*INFγ-R2 rs2834213*	Ab titer ≥ 2%	Ab titer ≥ 2%	Ab titer < 2%	Ab titer < 2%	OR	CI	*p* value *
AA	21	0.84	4	0.5	5.250	0.9115 to 30.238	0.0737
AG	3	0.12	4	0.5	0.1364	0.02170 to 0.8567	0.0418
GG	1	0.04	0	0	1.041		NS
*Il-6-174 rs1800796*	Ab titer ≥ 2%	Ab titer ≥ 2%	Ab titer < 2%	Ab titer < 2%	OR	CI	*p* value *
CC	12	0.48	0	0	15.741	0.8199 to 302.20	0.0299
CG	8	0.32	5	0.625	0.2824	0.05368 to 1.485	NS
GG	5	0.20	3	0.375	0.4167	0.07349–2.362	NS

* By Fisher’s exact test.

**Table 3:** Association between titers of IgM and IgG anti-B of group A, above or equal to the median and below the median, and cytokine polymorphisms.

Blood Group A							
**IgM anti-B**							
*IL-10-819 rs1800872*	Ab titer ≥ 8%	Ab titer ≥ 8%	Ab titer < 8%	Ab titer < 8%	OR	CI	*p* value *
CC	15	0.79	7	0.37	6.429	1.516–27.254	**0.0201**
CT	4	0.21	10	0.53	0.2400	0.05777–0.9971	0.0911
TT	0	0	2	0.10	0.1795	0.008046–4.004	NS
**IgG anti-B**							
*IL-18-137 rs187238*	Ab titer ≥ 0.5%	Ab titer ≥ 0.5%	Ab titer < 0.5%	Ab titer < 0.5%	OR	CI	*p* value *
GG	16	0.084	11	0.61	3.394	0.7164–16.080	NS
GC	1	0.05	7	0.39	0.08730	0.009424–0.8087	0.0188
CC	2	0.11	0	0	5.286	0.2365–118.12	NS

* By Fisher’s exact test.

**Table 4:** Association between titers of IgM and IgG anti-B of group B, above or equal to the median and below the median, and cytokine polymorphisms.

Blood Group B							
**IgM anti-A**							
*IL-18-607 rs1946518*	Ab titer ≥ 8%	Ab titer ≥ 8%	Ab titer < 8%	Ab titer < 8%	OR	CI	*p* value *
GG	14	0.625	7	1	0.08406	0.004329–1.632	0.0664
GT	10	0.312	0	0	10–161	0.5220–197.81	0.0694
TT	1	0.063	0	0	0.9184	0.03373–25.006	NS
**IgG anti-A**							
*INFGR2 rs2834213*	Ab titer ≥ 2%	Ab titer ≥ 2%	Ab titer < 2%	Ab titer < 2%	OR	CI	*p* value *
AA	21	0.84	4	0.5	5.250	0.9115–30.238	0.0737
AG	3	0.12	4	0.5	0.1364	0.02170–0.8567	0.0418
GG	1	0.04	0	0	1.041	0.3858–28.083	NS
*IL-6-174 rs1800796*	Ab titer ≥ 2%	Ab titer ≥ 2%	Ab titer < 2%	Ab titer < 2%	OR	CI	*p* value *
CC	12	0.48	0	0	15.741	0.8199–302.20	0.0299
CG	8	0.32	5	0.625	0.2824	0.05368–1.485	NS
GG	5	0.20	3	0.375	0.4167	0.07349–2.363	NS

* By Fisher’s exact test.

**Table 5:** Association between titers of all anti-A and -B IgG, above or equal to the median and below the median, and cytokine polymorphisms.

**Anti-A IgG**							
Polymorphism	Ab titer ≥ 4%	Ab titer ≥ 4%	Ab titer < 4%	Ab titer < 4%	OR	C.I	*p* value *
*INFγ-R2 rs2834213*							
AA	38	0.83	11	0.48	5.182	1.693 to 15.859	0.0045
AG	6	0.13	12	0.53	0	0.04200 to 0.4501	0.0010
GG	2	0.04	0	0	0	0.1216 to 57.336	NS
*IL-10 rs3021097*							
CC	19	0.41	16	0.70	0.3079	0.1061 to 0.8929	0.0406
CT	24	0.52	6	0.26	3.091	1.033 to 9.250	0.0446
TT	3	0.07	1	0.04	1.535	0.1506 to 15.639	NS
*IL-6 rs1800795*							
CC	20	0.435	4	0.17	3.654	1.072 to 12.5451	0.0362
CG	14	0.304	14	0.61	0.2813	0.09872 to 0.8013	0.0203
GG	12	0.261	5	0.22	1.271	0.3866 to 4.175	NS
**Anti-B IgG**							
Polymorphism	Ab titer ≥ 2%	Ab titer ≥ 2%	Ab titer < 2%	Ab titer < 2%	OR	CI	*p* value *
*IL-18-137 rs187238*							
GG	37	0.76	20	0.50	3.083	1.255 to 7.577	0.0155
GC	9	0.18	19	0.47	0.2487	0.09588 to 0.6450	0.0054
CC	3	0.06	1	0.03	2.543	0.2541 to 25.461	NS
*IL-4 rs2243250*							
CC	19	0.39	22	0.55	0.5182	0.2220 to 1.210	NS
CT	28	0.57	14	0.35	2.600	1.092 to 6.188	0.0342
TT	2	0.04	4	0.1	0.3830	0.06639 to 2.209	NS
*IFN-γ rs2430561*							
AA	31	0.63	23	0.575	1.273	0.5415 to 2.993	NS
AT	16	0.33	10	0.250	1.455	0.5726 to 3.695	NS
TT	2	0.04	7	0.175	0.2006	0.03916 to 1.028	0.0725

* By Fisher’s exact test.

**Table 6:** Association between titers of all anti-A and -B IgM, above or equal to the median and below the median, and cytokine polymorphisms.

**Anti-A IgM**							
Polymorphism	Ab titer ≥ 8%	Ab titer ≥ 8%	Ab titer < 8%	Ab titer < 8%	OR	CI	*p* value *
*IL-18-607 rs1946518*							
GG	27	0.51	13	0.86	0.1598	0.03279 to 0.7784	0.0170
GT	23	0.43	1	0.07	10.733	1.313 to 87.715	0.0124
TT	3	0.06	1	0.07	0.8400	0.08091 to 8.721	NS
**Anti-B IgM**							
Polymorphism	Ab titer ≥ 8%	Ab titer ≥ 8%	Ab titer < 8%	Ab titer < 8%	OR	CI	*p* value *
*INFγ-R2 rs2834213*							
AA	30	0.623	23	0.885	0.2174	0.05705 to 0.8284	0.0292
AG	16	0.333	3	0.115	3.833	0.9989 to 14.710	0.0524
GG	2	0.042	0	0	2.849	0.1317 to 61.652	NS
*IL-10 rs3021097*							
CC	32	0.67	9	0.35	3.778	1.380 to 10.338	0.0137
CT	15	0.31	14	0.54	0.3896	0.1457 to 1.042	0.0811
TT	1	0.02	3	0.11	0.1631	0.01606 to 1.657	NS
*IL-1 R1 rs2234650*	Ab titer ≥ 8%	Ab titer ≥ 8%	Ab titer < 8%	Ab titer < 8%	OR	CI	*p* value *
CC	26	0.54	21	0.81	0.2814	0.09101 to 0.8700	0.0259
CT	20	0.42	5	0.19	3.000	0.9673 to 9.304	0.0719
TT	2	0.04	0	0	2.849	0.1317 to 61.652	NS

* By Fisher’s exact test.

**Table 7:** Univariate correlations between the titer of all anti-A and anti-B IgG antibodies, respectively, and demographic factors and circulating cytokines in donors.

	**Anti-A IgG**	
**Variables**	**r Values**	*p* Values *
Age	−0.16	0.01
Males	0.22	>0.05
Females	0.33	>0.05
Il-18	0.24	0.049
IL-1α	0.31	>0.05
IL-1β	0.41	>0.05
IL-6	0.29	0.03
IL-4	0.28	0.03
IL-1R1	0.24	0.021
IL-13	0.48	0.05
TNFα	0.57	>0.05
IFN-γ	0.29	0.01
IL-10	−0.38	0.04
**Anti-B IgG**		
**Variables**	**r Values**	*p* Values *
Age	−0.21	0.02
Males	0.29	>0.05
Females	0.39	>0.05
Il-18	0.29	>0.05
IL-1α	0.35	>0.05
IL-1β	0.55	>0.05
IL-6	0.22	0.02
IL-4	0.23	0.03
IL-1R1	0.31	0.04
IL-13	0.51	>0.05
TNFα	0.41	>0.05
IFN-γ	0.23	0.01
IL-10	−0.22	0.03

* By linear Pearson correlation test, or non-parametrical Spearman correlation test, when appropriate.

**Table 8:** Univariate and multivariate regression analysis of demographic factors, SNPs, and circulating cytokines associated with titers of all anti-A and anti-B IgG antibodies in the study population.

Variables	Unadjusted OR (95%CI)	*p*	Adjusted OR (95%CI) *	*p*
Age	1.02 (1.01–1.05)	0.02	1.01 (0.98–1.06)	0.011
IL-6	1.27 (0.99–2.4)	0.01	1.15 (0.69–1.78)	0.041
IL-4	1.32 (0.91–1.35)	0.039	2.01 (1.02–2.1)	0.02
IL-1R1	1.78 (0.89–1.99)	0.12	-	-
IL-10	1.4 (1.3–2.21)	0.02	1.98 (1.67–2.1)	0.039
INF-γ IL-6rs1800795C/IL-4rs2243250T/IL-10rs3021097T/IFN-γ rs2430561T combined genotype_profile	2.51 (0.8–3.1) 1.39 (0.99–2.4)	0.01 0.04	2.1 (1.2–2.98) 1.59 (1.67–2.1)	0.058 0.042

* Adjusted for age, IL-6, IL-1R1, INF-γ, and IL-10. The Hosmer–Lemeshow test statistic was 7.12 (df = 7.9, *p* = 0.52), which revealed good model fit.

## Data Availability

The original contributions presented in this study are included in the article. Further inquiries can be directed to the corresponding author.

## Supplementary Materials

The following supporting information can be downloaded at: https://www.mdpi.com/article/10.3390/ijms27083629/s1.
