# Supplementary material for: Cytokine Gene Polymorphisms Modulate Isohemagglutinin Titers and Classes: Another Aspect Towards the Link Between ABO Groups and Human Pathologies?
Source: Int J Mol Sci. 2026 Apr 18;27(8):3629. doi: 10.3390/ijms27083629 (PMC13116671; doi:10.3390/ijms27083629)
Supplement: Supplementary file 1 [file ijms-27-03629-s001.zip › ijms-4199861-supplementary.pdf]

Table S1: **Titration of total isohaemagglutinins of the IgM anti-A, anti-B and IgG anti-A and anti-B classes in all the blood samples from 108 donors enrolled in the study**

TOTAL	IgM anti-A	IgM anti-B	IgG anti-A	IgG anti-B
**Mean**	14,014	12,8	49,12	22,01
**SD**	12,562	18,48	76.202	42,680
**STD**	1,523	2,106	9,174	4,451
**Sample size**	48	55	57	53
**Lower 95% Conf. limit**	10,972	8,630	22,257	13,156
**Upper 95% Conf. limit**	17,058	17,033	58,902	30,886
**Minimum**	0,00	0,00	0,00	0,00
**Median**	8	8	4	2
**Maximum**	64,00	128,00	512,00	256,00
**Normality test KS**	0,2755	0,3280	0,2827	0,3351
**Normality test P value**	<0.0001	<0.0001	<0.0001	<0.0001

Table S2: Titration of isohaemagglutinins, IgM and IgG anti-B in the blood samples from donors with A group.

Blood Group A	IgM anti-B	IgG
Mean	8,048	4,189
SD	6,348	11,088
Sample size	41	40
STD	0,9914	1.753
Lower 95% Conf. limit	6,045	0,5283
Upper 95% Conf. limit	10,052	7,622
Minimum	2,00	0
Median	8,00	0,5
Maximum	32,00	64
Normality test KS	0,2592	0,3777
Npormality test p value	0,0081	<0,001

Table S3: Titration of isohaemagglutinins, IgM and IgG anti-A in the blood samples from donors with group B.

Blood Group B	IgM anti-A	IgG anti-A
Mean	12,83	6,8
SD	10,100	12,713
Sample size	33	33
STD error of mean	1,758	2,213
Lower 95% Conf. limit	9,417	1,914
Upper 95% Conf. limit	16,583	10,934
Minimum	1,00	0
Median	8,00	2,00
Maximum	32,00	64,00
Normality test KS	0,2958	0,3938
Npormality test p value	0,0062	<0,001

Table S4: Titration of isohaemagglutinins, IgM and IgG anti-A and anti-B in the blood samples from donors with O group.

Blood Group 0	IgM anti-A	IgM anti-B	IgG anti-A	IgG anti-B
Mean	15,86	20,86	79,8	54,27
SD	14,577	25.606	96,996	55.870
Sample size	34	30	34	33
STD error of mean	2,2500	4.391	16.635	9.726
Lower 95% Conf. limit	10,323	10.413	40.372	36.605
Upper 95% Conf. limit	20,501	28.293	108.10	76.243
Minimum	4,00	2,00	0	2,00
Median	12,00	8,00	64	32
Maximum	64,00	128	512	256
Normality test KS	0,3368	0,3462	0,2479	0,2340
Npormality test p value	0,0009	0,0006	0,0306	0,0540

Table S5: Titration of total isohaemagglutinins, IgM and IgG anti-A and anti-B in the blood samples from male donors

Males	IgM anti-A	IgM anti-B	IgG anti-A	IgG anti-B
Mean	12,625	8,816	28,73	16,09
SD	12,471	9,941	39,909	29,694
Sample size	40	40	41	57
STD error of mean	1,972	1,420	6,233	3,933
Lower 95% Conf. limit	8,636	5,598	16,135	8,205
Upper 95% Conf. limit	16,614	11,674	41,328	23,970
Minimum	0	0	0	0
Median	8	8	4	2
Maximum	64	64	128	128
Normality test KS	0,2696	0,3286	0,2836	0,3442
Npormality test p value	0,0060	<0,0001	0,0027	<0,0001

Table S6. Titration of total isohaemagglutinins, IgM and IgG anti-A and anti-B in the blood samples from female donors

Female	IgM anti-A	IgM anti-B	IgG anti-A	IgG anti-B
Mean	16	19,85	57,9	31,65
SD	12,649	26,561	10,835	57,190
Sample size	28	28	28	35
STD error of mean	2,390	5.019	20,476	9,667
Lower 95% Conf. limit	11,095	9,557	15,912	11,999
Upper 95% Conf. limit	20,920	30.157	99,945	51,316
Minimum	4,00	2,00	0	0
Median	16,00	8,00	6,00	4,00
Maximum	64,00	128	512	256
Normality test KS	0.3214	0,3434	0,2847	0,3222
Npormality test p value	0.00061	0,0027	0,0214	0,0014

Table S7: Results of statistical analysis between total IgM anti-A and anti-B titre and cytokine polymorphisms in male subjects.

IgG anti-A MALES							
Polymorphism	Ab titer ≥4	Ab titer ≥4 %	Ab titer <4	Ab titer <4 %	OR	CI	P value
INFGRB *rs2430561*							
AA	24	0.89	7	0,50	8,000	1,626-39,369	**0,0175**
AG	3	0.11	7	0,50	0,1250	0,02540-0,6151	**0,0175**
GG	0	0	0	0	0		NS
IgG anti-B							
Il-8-137 *rs187238*	Ab titer ≥2	Ab titer ≥2 %	Ab titer <2	Ab titer <2 %	OR	CI	P value
GG	23	0,80	13	0,5	3,833	1.175-12,570	**0,0272**
GC	3	0,10	13	0,5	0,1154	0,02785-0,4780	**0,0023**
CC	3	0,10	0	0	3.566	1.1713-74.218	NS
IL-10-592 rs*3021097*							
CC	10	0,34	17	0,65	0,2786	0,09151-0,8484	**0,0315**
CA	16	0,55	8	0,31	2,769	0,9136-8,394	NS
AA	3	0,10	1	0,04	2,885	0,2808-29,630	NS

Table S8: Results of statistical analysis between total IgG anti-A and anti-B titre and cytokine polymorphisms in male subjects

Males IgM anti-A							
Polymorphism	Ab titer ≥8	Ab titer ≥8 %	Ab titer <8	Ab titer <8 %	OR	CI	P value
Il-10 -819 rs1800872							
CC	5	0,19	8	0,62	0,1430	0,03231-0,6244	**0,0114**
CT	19	0,70	5	0,38	3,800	0,9466-15,255	0,0857
TT	3	0,11	0	0	3,857	0,1850-80,433	NS
IgM anti-B							
IL-1 -511 *rs16944*	Ab titer ≥8	Ab titer ≥8 %	Ab titer <8	Ab titer <8 %	OR	CI	P value
AA	0	0	3	0,15	0,09091	0,004419 -1,870	0,0703
AG	12	0,44	8	0,40	1,200	0,3711-3,880	NS
GG	15	0,56	9	0,45	1,528	0,4774-4,889	NS

.

Table S9: Results of statistical analysis between total anti-B and anti A IgM titre and cytokine polymorphisms in female subjects

**IgM anti A Females**							
**Polymorphism**	Ab titer ≥16	Ab titer ≥16 %	Ab titer <16	Ab titer <16 %	OR	CI	P value
INF gamma *rs 2430561*							
AA	7	0,47	12	0,92	0,07292	0,007467-0,7121	**0,0157**
AT	8	0,53	0	0	30,600	1,540-608,09	**0,025**
TT	0	0	1	0,08	0,2688	0,01004-7,194	NS
IgM anti B							
Polymorphism	Ab titer ≥8	Ab titer ≥8%	Ab titer <8	Ab titer <8%	OR	CI	P value
INFGRB2 *rs2834213*							
AA	11	0,52	6	1	0,08425	0,004210-1,686	NS
GA	9	0,43	0	0	9,880	0,4927-198,12	0,0707
GG	1	0,05	0	0	0,9512	0,03436-26,332	NS

Table S10: Results of statistical analysis between total anti-A and anti-B IgG titre and cytokine polymorphisms in female subjects

IgG Anti-A FEMALES							
Polymorphism	Ab titer ≥**6**	Ab titer ≥**6**%	Ab titer <**6**	Ab titer <**6**%	OR	CI	P value
Il-13-1055 *rs1800925*							
CC	13	0,93	8	0,57	9,750	0,9839-96,615	0,0768
CT	1	0,007	6	0.43	0,1026	0,01035-1.016	0,0768
TT	0	0	0	0	0		NS
IgG anti.B							
Il-1-511 *rs16944*	Ab titer ≥4	Ab titer ≥4%	Ab titer <4	Ab titer <4%	OR	CI	P value
AA	1	0,05	13	0,87	0,01385	0,02430-0,7891	**0,0297**
AG	9	0,47	2	0,13	5.850	1.026-33,340	0,0640
**GG**	9	0,47	0	0	2.514	0,09536-66,253	NS
